# Optical microscopic study on a novel morphological classification method of multiple diagnostic features of *Sarcoptes scabiei* var. *hominis*

**DOI:** 10.1017/S0031182023000938

**Published:** 2023-09

**Authors:** Wanchen Li, Xiaoli Li, Lin Song, Hongfeng Li, Yaliu Wu, Jianjun Li

**Affiliations:** 1 Department of Clinical Laboratory, The Second Hospital of Tianjin Medical University, Tianjin, PR China; 2 Department of Clinical Laboratory, Tianjin Academy of Traditional Chinese Medicine Affiliated Hospital, Tianjin, PR China

**Keywords:** morphological classification, optical microscopy, *Sarcoptes scabiei* var. *hominis*, scabies

## Abstract

Optical microscopy is the gold standard technique used to confirm the diagnosis of scabies. Multiple diagnostic features of the pathogen *Sarcoptes scabiei* var. *hominis* (*S. scabiei*) can be identified under a microscope and classified into 3 categories: mites, eggs and fecal pellets. However, mite and eggshell fragments can also be observed, which have been ignored in the 2020 International Alliance for the Control of Scabies (IACS) Criteria and by most researchers. In this study, we propose a novel morphological classification method that classifies multiple diagnostic features into 5 categories and 7 subcategories. Our results revealed that 65.2% (1893 of 2896) of the positive cases were confirmed through the identification of mites, eggs or fecal pellets, whereas up to 34.6% (1003 of 2896) of the positive cases were confirmed through the identification of mite or eggshell fragments. Therefore, the important diagnostic values of mite and eggshell fragments should be emphasized. Importantly, for the first time, mite and eggshell fragments were classified into 7 subcategories, some of which are easily ignored or confused with contaminating artefacts. We believe that this novel morphological classification method will be beneficial for operator training in interpreting slides and in improving the 2020 IACS Criteria.

## Graphical abstract

**Figure:**
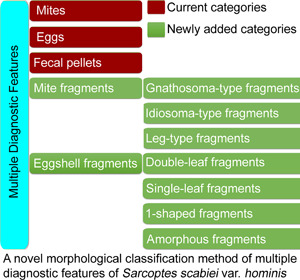

## Introduction

Scabies is a contagious skin disease caused by the pathogen *Sarcoptes scabiei* var. *hominis* (*S. scabiei*) and is estimated to affect approximately 200 million people globally (GBD 2015 Disease and Injury Incidence and Prevalence Collaborators, 2016; Leung *et al*., 2020). Although the earliest written reference to scabies appeared in Leviticus in the Bible (1200 BCE) (Roncalli, 1987), the lack of standardized diagnostic methods has persisted. Clinically, common scabies is characterized by pruritus and multiple skin lesion morphologies, whereas crusted scabies most frequently occurs in immunocompromised patients and presents with hyperkeratosis with or without pruritus (Thomas *et al*., 2020). However, these manifestations are non-specific and mimic those of other skin diseases such as atopic dermatitis, eczema, psoriasis, diaper rash, insect bites and poison ivy (Arlian and Morgan, 2017). A presumptive diagnosis of scabies may be made based on clinical manifestations; however, scabies is commonly misdiagnosed. A single-centre retrospective study in the United States reported that 45.3% of patients with scabies were previously misdiagnosed (Anderson and Strowd, 2017).

To address this issue, the International Alliance for the Control of Scabies (IACS) led a project to develop consensus criteria for the diagnosis of common scabies in 2018 (Engelman *et al*., 2018), and released the latest consensus standards in 2020 (Engelman *et al*., 2020). The 2020 IACS Criteria comprise 3 levels: confirmed, clinical and suspected scabies. Optical (light) microscopy of skin scrapings is considered the gold standard technique because it can reveal multiple diagnostic features of *S. scabiei* to support the diagnosis of confirmed scabies. However, it has several disadvantages such as insensitivity, operator dependence, time consumption and invasive operation, and its accuracy depends on the expertise of the operator (Engelman *et al*., 2020).

As described in the 2020 IACS Criteria and in most papers (Nonaka *et al*., 1985; Walton and Currie, 2007; Ghosh *et al*., 2017; Lydeamore *et al*., 2019; Nelson *et al*., 2019; Bernigaud *et al*., 2020; Engelman *et al*., 2020), diagnostic features are classified into 3 categories: mites (adults and immature forms), eggs and fecal pellets (scybala). However, a complete mite may be cut into fragments by the sterile blade in the process of extracting materials. Eggshell fragments can also be observed, some of which are easily ignored or confused with contaminating artefacts. However, the important diagnostic values of mite and eggshell fragments have been ignored by the 2020 IACS and most researchers.

Therefore, a novel morphological classification method is proposed, in which multiple diagnostic features of *S. scabiei* are classified into 5 categories and 7 subcategories. The method was discussed in detail. Here, the important diagnostic values of mite and eggshell fragments were emphasized. We believe that this novel morphological classification method will be beneficial for operator training in interpreting slides and in improving the 2020 IACS Criteria.

## Materials and methods

### Data collection

This study was conducted at the Tianjin Academy of Traditional Chinese Medicine Affiliated Hospital, in Tianjin, China. Tianjin is the second largest city in northern China. Most of the subjects in this study come from Tianjin, with a small portion coming from other regions of China. Microscopic images and the associated results from subjects who underwent optical microscopy examination of skin scrapings for the diagnosis of scabies between 2018 and 2022 were retrieved. This study did not require personal information from patients. It was approved and monitored by the hospital ethics committee, which approved an exemption from obtaining patient consent.

### Subjects

The subjects who underwent optical microscopy examination were selected by clinicians based on clinical evaluation, including the following individuals:

1. Individuals with confirmed scabies, who required an efficacy evaluation after treatment;
2. Individuals with clinical or suspected scabies, who required a diagnosis of confirmed scabies;
3. Individuals with other skin diseases, who required a differential diagnosis from scabies;
4. Individuals with a history of contact with other individuals with confirmed scabies, who required to determine whether they were infected with scabies.

### Morphological classification method of multiple diagnostic features of *S. scabiei*

The diagnostic features of *S. scabiei* observed under an optical microscope were classified into 5 categories and 7 subcategories.

**Figure 1.**
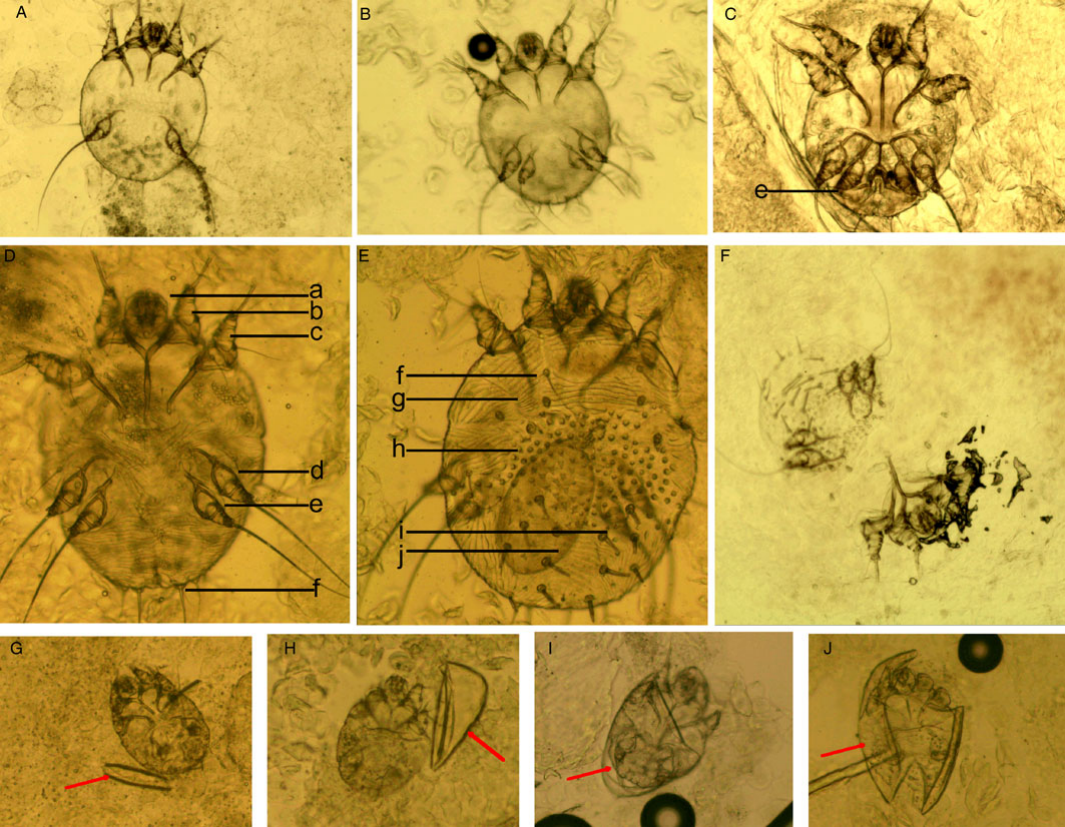
Examples of mites and the anatomic structures. (A) Larvae; (B) nymphs; (C) adult male mites; (D) adult female mites (ventral aspect); (E) adult female mites (dorsal aspect); (F) a mite was cut into 2 parts; however, the gnathosoma and most idiosoma structures can be observed in a field; （G-J） larva with incomplete eggshells (red arrow). Complete body structures: a, gnathosoma; b-d, legs Ⅰ, Ⅱ, Ⅲ, and Ⅳ; f, long bristles; g, cuticular striations; h, cuticular spines; i, setae; j, ovum (magnification: 100).

**Figure 2.**
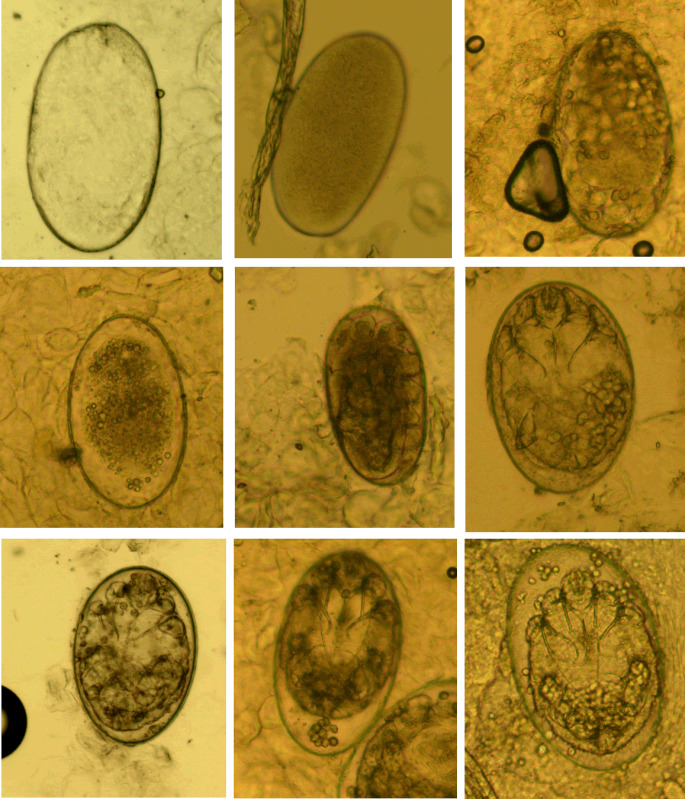
Eggs at different developmental levels (magnification: 100).

**Figure 3.**
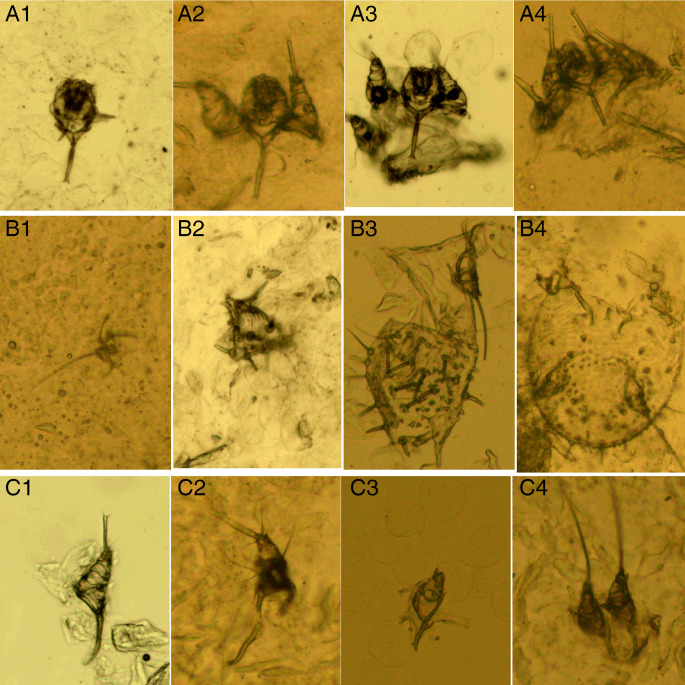
Mite fragments. (A1–A4) Gnathosoma-type fragments; (B1–B4) idiosoma-type fragments; (C1–C4) leg-type fragments (magnification: 100).

**Figure 4.**
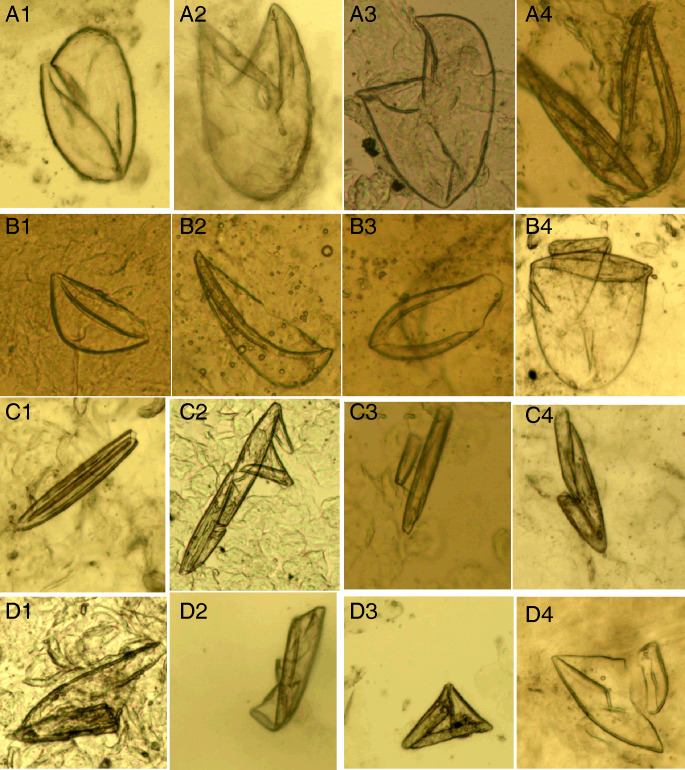
Eggshell fragments. (A1–A4) Double-leaf fragments; (B1–B4) single-leaf fragments; (C1–C4) 1-shaped fragments; (D1–D4) amorphous fragments (magnification: 100).

- *Mites*. The developmental stages of *S. scabiei* include eggs, larvae, nymphs (protonymphs and tritonymphs) and adults (Lydeamore *et al*., 2019). In this study, mites refer to immature (larvae and nymphs) and mature (adult) forms (Fig. 1A–E). The anatomical structures of a mite include a gnathosoma and an idiosoma; the idiosoma bears setae, cuticular spines, cuticular striations, long bristles and 3 (larvae) or 4 (nymphs and adults) pairs of legs (Nonaka *et al*., 1985; Walton and Currie, 2007; Nelson *et al*., 2019) (Fig. 1A–E). Most of the anatomical structures of a mite should be complete. In some cases, a mite can be cut into several parts by a sterile blade in the process of extracting materials; however, the gnathosoma and most idiosoma structures should be observed in the same field under an optical microscope (Fig. 1F). Note that the eggshells of larvae should be either absent or incomplete (Fig. 1G–J). Examples and the anatomical structures are shown in Fig. 1.
- *Eggs*. Eggs refer to ova or eggs with complete eggshells. A complete eggshell is the key characteristic of this category (Fig. 2).
- *Mite fragments*. Mite fragments are incomplete structures of mites cut using a sterile blade (Fig. 3). This category includes 3 subcategories: gnathosoma-type, idiosoma-type and leg-type fragments. Gnathosoma-type fragments refer to incomplete structures with gnathosoma (Fig. 3 A1–A4); whereas idiosoma-type fragments refer to incomplete structures without gnathosoma (Fig. 3 B1–B4); and leg-type structures are incomplete structures with only legs (Fig. 3 C1–C4). In contrast to mites, the gnathosoma and most body structures of idiosoma should not be observed in the same field under an optical microscope.
- *Eggshell fragments*. Eggshell fragments are incomplete eggshells left by eggs after hatching into larvae. As shown in Fig. 4, the eggshell fragments exhibited different shapes. Based on the differences in size and shape, this category can be classified into 4 subcategories: double-leaf, single-leaf, 1-shaped and amorphous fragments.

Double-leaf fragments are relatively large. In some cases, the eggshell is slightly damaged with only 1 chink (Fig. 4 A1); in most cases, the eggshell is broken into 2 connected fragments, shaped like the letter ‘V’ (Fig. 4 A2–A4).

Single-leaf fragments are smaller than the double-leaf fragments. The eggshell is broken into 2 unconnected fragments shaped like a boat, helmet or pan (Fig. 4 B1–B4).

If an eggshell is severely damaged, several small fragments will remain. Some small fragments are shaped like the Arabic numeral ‘1’, which are referred to as 1-shaped fragments (Fig. 4 C1–C4); some irregular small fragments are referred to as amorphous fragments (Fig. 4 D1–D4). Note that the 1-shaped and amorphous fragments are easily confused with contaminating artefacts.

**Figure 5.**
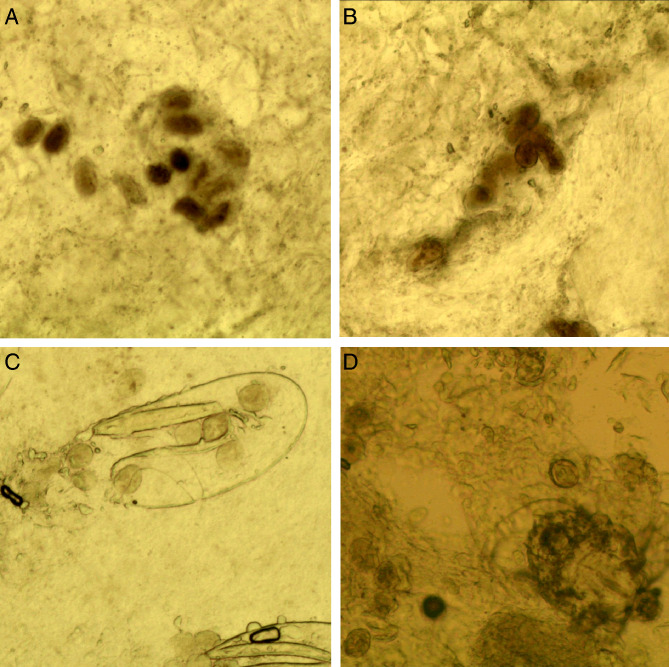
Fecal pellets. (A and B) Fecal pellets in piles; (C) fecal pellets and a double-leaf fragment; (D) fecal pellets and a mite (magnification: 100).

- *Fecal pellets (scybala)*. Fecal pellets are the products of mites and are seen as small, oval structures that are easily confused with contaminating artefacts. Therefore, fecal pellets must appear in piles (Fig. 5).

### Optical microscopy examination

The examination was performed by operators with more than 3 years of clinical experience as described elsewhere (Engelman *et al*., 2020). Firstly, several suspected sites were determined based on multiple skin lesion morphologies involving finger web-spaces, hands, the volar surfaces of the wrists, axillae, buttocks, the areola in women, the toe seams in infants or young children and genitalia in men. Following that, skin scrapings were collected from the suspected sites on the same individual using a sterile blade (#15) after disinfection with 75% alcohol. The scrapings were then carefully placed on a standard glass microscope slide, mixed with 1–2 drops of potassium hydroxide (KOH) at a 15% concentration, and covered with a coverslip. The slide was left to stand for several minutes before being scanned under a microscope (Olympus CX33; Tokyo, Japan) at 100× magnification to search for the diagnostic features of *S. scabiei*. Finally, a field was selected according to the priority order of diagnostic features stated as follows, and then saved as a microscopic image using a camera (JEDA SmartVF650DO, Jiangsu, China) along with its automated imaging system.

### Priority order of diagnostic features of *S. scabiei*

Some diagnostic features (mites and eggs) are easy to identify, whereas others (mite and eggshell fragments, and fecal pellets) may be difficult to identify or easily confused with contaminating artefacts. Meanwhile, multiple diagnostic features can be observed in a single examination. In this study, the priority order of diagnostic features is as follows: (1) mites; (2) eggs; (3) double-leaf fragments; (4) leg-type fragments; (5) single-leaf fragments; (6) gnathosoma-type fragments; (7) idiosoma-type fragments; (8) 1-shaped fragments; (9) amorphous fragments; (10) fecal pellets.

### Positive detection rates

In this study, a positive case refers to an optical microscopy examination in which at least 1 diagnostic feature of *S. scabiei* was observed, whereas a negative case refers to an optical microscopy examination in which no diagnostic features of *S. scabiei* were observed. The annual and overall numbers of positive cases and total cases between 2018 and 2022 were counted. The annual and overall positive detection rates were then calculated as shown in equation (1).1
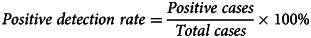


### Frequency distribution of various diagnostic features of *S. scabiei*

According to the priority order of diagnostic features, all positive cases between 2018 and 2022 were divided into 10 groups: mites, eggs, double-leaf fragments, leg-type fragments, single-leaf fragments, gnathosoma-type fragments, idiosoma-type fragments, 1-shaped fragments, amorphous fragments, and fecal pellets. The numbers of positive cases in each group *n_i_* (*i* = 1, …, 10) and the total positive cases were counted. The frequency of each group was then calculated as shown in equation (2).2
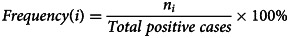


### Statistical analysis

All statistical analyses were performed using Microsoft Excel. Positive detection rates and frequencies were used for descriptive analysis, and the statistical results of the descriptive studies were directly compared.

## Results

### Positive detection rates

A total of 23 244 cases were retrieved from our database between 2018 and 2022: 4459 cases in 2018, 4813 cases in 2019, 3643 cases in 2020, 4909 cases in 2021 and 5420 cases in 2022. Of these cases, 2896 were confirmed to be positive, with an overall positive detection rate of 12.5% (2018–2022). The positive detection rate in 2019 was the highest (15.9%, 764 of 4813), followed by 2018 (14.6%, 653 of 4459), 2020 (13.5%, 490 of 3643), 2021 (11.0%, 542 of 4909) and 2022 (8.3%, 447 of 5420) (Table 1).

**Table 1.** Positive detection rates between 2018 and 2022

Year	Total cases (*n*)	Positive cases (*n*)	Positive detection rates (%)
2018	4459	653	14.6
2019	4813	764	15.9
2020	3643	490	13.5
2021	4909	542	11.0
2022	5420	447	8.3
Total	23 244	2896	12.5

### Frequency distribution of various diagnostic features of *S. scabiei*

Of the 2896 positive cases, 1193 (41.2%) were confirmed through the identification of mites, followed by eggs (23.9%, 691 cases), eggshell fragments (19.2%, 556 cases), mite fragments (15.4%, 447 cases) and fecal pellets (0.3%, 9 cases). Importantly, 34.6% of the positive cases (1003 of 2896) were confirmed through the identification of eggshell or mite fragments. Specifically, 250 cases (8.6%) were confirmed through the identification of double-leaf fragments, followed by leg-type fragments (6.4%, 185 cases), single-leaf fragments (5.4%, 156 cases), gnathosoma-type structures (4.7%, 136 cases), idiosoma-type structures (4.4%, 126 cases), 1-shaped fragments (3.4%, 99 cases) and amorphous fragments (1.8%, 51 cases). More detailed information is provided in Table 2.

**Table 2.** Frequency distribution of various diagnostic features of *S. scabiei* between 2018 and 2022

Diagnostic features	Frequency [*n* (%)]
A. Mites	1193(41.2)
B. Eggs	691(23.9)
C. Eggshell fragments	556(19.2)
C1. Double-leaf fragments	250(8.6)
C2. Single-leaf fragments	156(5.4)
C3. 1-shaped fragments	99(3.4)
C4. Amorphous fragments	51(1.8)
D. Mite fragments	447(15.4)
D1. Gnathosoma-type fragments	136(4.7)
D2. Idiosoma-type fragments	126(4.4)
D3. Leg-type fragments	185(6.4)
E. Fecal pellets	9(0.3)
Total	2896(100.0)

A, B, C, D, and E: 5 categories of diagnostic features; C1, C2, C3, and C4: 4 subcategories of eggshell fragments; D1, D2, and D3: 3 subcategories of mite fragments.

## Discussion

Optical microscopy is considered the gold standard for diagnosing scabies (Arora *et al*., 2020). Multiple diagnostic features of *S. scabiei* can be identified using microscopy. In this study, the diagnostic features were classified into 5 categories. Our results revealed that 65.2% of the positive cases were determined through the identification of mites, eggs or fecal pellets. Importantly, up to 34.8% of the positive cases were determined through the identification of mite or eggshell fragments. This result proved the important diagnostic values of mite and eggshell fragments.

Conflicting views regarding the diagnostic value of fecal pellets have been reported. Most researchers affirmed the diagnostic value of fecal pellets (Anderson and Strowd, 2017; Arlian and Morgan, 2017; Engelman *et al*., 2018; Engelman *et al*., 2020; Thomas *et al*., 2020); whereas others considered that the presence of fecal pellets should not be considered diagnostic, as they resemble artefacts or debris (Arora *et al*., 2020). Our results showed that only 0.3% (9 of 2896) of the positive cases were confirmed only through the identification of fecal pellets. We agree with the diagnostic value of fecal pellets. We suggest that fecal pellets must appear in piles and that a positive conclusion requires the unanimous consent of 2 experienced operators.

One disadvantage of optical microscopy is operator dependence – training and expertise are required to find lesions, extract material and prepare and interpret slides (Engelman *et al*., 2020). Generally, the diagnostic features of *S. scabiei* can be observed in skin scrapings from lesions around the fingers, hands, wrists, infant toe seams and male genitalia. Some researchers think that lesions on the trunk most commonly develop from hypersensitivity reaction and cannot find mites of *S. scabiei* (Engelman *et al*., 2020); however, it applies to small lesions such as papules, but not to big lesions such as nodules. In fact, diagnostic features can be observed in scrapings from nodules around the armpit and buttocks (data not shown). It should be noted that nodules (especially those on male genitalia) can be long-term and it is difficult to observe diagnostic features from skin scrapings obtained around the nodules after treatment.

Another disadvantage of microscopy is insensitivity (Engelman *et al*., 2020). One study reported that the sensitivity of optical microscopy was 0.46 (95% CI 0.31–0.62) (Walter *et al*., 2011). A study in Elazığ reported that the overall positive detection rate was 18.6% (139 of 746) (Yücel and Yılmaz, 2021). Another study in Accra, Ghana reported that 92 patients were diagnosed with clinical scabies, but all skin scrapings examined with optical microscopy turned out to be negative (Kaburi *et al*., 2019). In the current study, the overall positive detection rate over 5 years (2018–2022) was 12.5%; however, it should not be considered to be due to the sensitivity of optical microscopy. In this study, some examinations were conducted on individuals after treatment, or those with other skin diseases. In these cases, the results were usually negative.

There are several reasons why microscopy is insensitive. Firstly, the examination is invasive. Some subjects, especially infants and young children, may be poorly tolerated in the process of extracting materials, resulting in a failed or invalid sample collection. Secondly, the reagent used in sample processing is KOH solution, which can only degrade keratin and cannot increase the contrast between diagnostic features and sample. Recently, it has been reported that *S. scabiei* can produce fluorescence under the ultraviolet mode of the dermatoscope (Nie *et al*., 2021). This is of great significance for improving the sensitivity of microscopy.

This study has some limitations, as it is the first study to classify the diagnostic features of *S. scabiei* into 5 categories and 7 subcategories. Therefore, this morphological classification method and the related terminology should be optimized in the future, and we look forward to suggestions and discussions from other researchers.

Although all diagnostic features share the same diagnostic value, some features are difficult to identify or easily confused with contaminating artefacts. In some cases, only a long bristle or several cuticular spines were identified (Fig. 3 B1–B2), which may be ignored by inexperienced operators. To the best of our knowledge, few studies have focused on the morphological classification of multiple diagnostic features of *S. scabiei* under a microscope. This study may be beneficial for operator training. Importantly, we suggest that the IACS Criteria should be revised in the following version to emphasize the important diagnostic values of mite and eggshell fragments.

## Data Availability

The complete datasets are available upon reasonable request.
